# Inclusive seed systems for better nutrition and sustainable food systems in Mali

**DOI:** 10.3389/fnut.2025.1628431

**Published:** 2025-10-02

**Authors:** Almamy Sylla, Kavitha Kasala, Padmaja Ravula, Victor Afari-Sefa, Boubakary Cissé, Mamourou Sidibé, McDonald Bright Jumbo

**Affiliations:** ^1^International Crops Research Institute for the Semi-Arid Tropics (ICRISAT), Bamako, Mali; ^2^International Crops Research Institute for the Semi-Arid Tropics (ICRISAT), Patancheru, India; ^3^International Crops Research Institute for the Semi-Arid Tropics (ICRISAT), Kano, Nigeria

**Keywords:** seed systems, biofortification, women, nutrition, food systems, sustainability

## Abstract

Women are central to agricultural production in Mali, yet systemic barriers limit their participation in inclusive seed systems and resilient food systems. In seed systems, restricted access to high-quality seeds, financial constraints, and limited technical training hinder women’s engagement in formal seed production and distribution. Within food systems, weak market integration, inadequate mechanization, and insufficient processing infrastructure constrain their ability to transform biofortified crops into value-added food products. Addressing these challenges requires a holistic, gender-responsive approach that strengthens women’s roles across the seed-to-nutrition pathway. This perspective article synthesizes insights from two key initiatives: The Networking for Seed Project-Phase II, which enhances community seed systems by promoting women’s participation in seed production and dissemination, and a pilot initiative by the World Food Program, which facilitates the processing, commercialization, and market integration of biofortified crops. These initiatives highlight the importance of linking seed systems with food processing and market access to create economic opportunities for women while improving household and community nutrition. By enhancing financial inclusion, utilizing technological tools, and strengthening institutional support, these approaches help women shift from being passive recipients of agricultural inputs to becoming active and empowered entrepreneurs. Integrating women more effectively into both seed and food systems contributes to more sustainable and equitable agricultural value chains, strengthening food security, nutrition outcomes, and climate resilience in Mali.

## Introduction

Mali’s agriculture is largely rainfed, with smallholder farmers—especially women—at the heart of food production. Women play a vital role in cultivating staple crops, yet they face persistent barriers in accessing productive resources. Although the introduction of improved and biofortified crop varieties offers promise for reducing malnutrition, women’s involvement in the seed sector and value-added processing remains limited.

This perspective article examines how strengthening community seed systems can contribute to improved food security and nutrition. While existing literature acknowledges the nutritional potential of improved crop varieties, there is limited understanding of how inclusive seed systems—particularly those that actively engage women—can enhance adoption, utilization, and impact at the community level. By addressing this gap, the article contributes to ongoing efforts to make seed systems more equitable and nutrition-sensitive. Although several initiatives across sub-Saharan Africa have sought to modernize seed systems—such as farmer-managed seed enterprises and participatory varietal selection programs—many have struggled to achieve sustained impact without dedicated gender-responsive approaches (1, 2). Farmer seed networks in Mali are largely based on the self-production of millet and sorghum seeds, with exchanges occurring within non-commercial, community-based systems (3, 4). These networks help maintain genetic diversity but offer limited access to improved varieties and hybrids, which are crucial for climate adaptation, food and nutritional security (5). Seed system interventions in turn leads to higher yields, crop improvement, and better household welfare (6, 7). These experiences highlight the need to strengthen community seed systems in ways that are not only technically sound but also socially inclusive, ensuring that women are fully integrated into seed production, dissemination, and decision-making processes. By emphasizing women’s participation in seed production and the processing of biofortified crops, this article presents practical strategies to enhance their role in agricultural value chains and bridge the gap between seed access and the availability of nutrition-rich consumer products.

Seed systems can be broadly understood through three interconnected pathways: the formal, the informal, and the community-based. The formal pathway involves breeding programs, seed companies, and agro-dealers delivering improved seeds to farmers. The informal pathway remains vital in many rural contexts, where farmer-saved seed, exchanges among relatives and neighbors, and local markets continue to dominate. The community-based pathway brings in research and extension support, with seed banks, cooperatives, and farmer groups playing key roles in distribution. To make these pathways more inclusive, four cross-cutting components are essential: improving access through targeted training and recognition of women’s traditional seed-keeping roles; enhancing affordability via subsidies, micro-credit, and savings groups; strengthening physical accessibility through localized distribution and women-led outlets; and increasing participation by ensuring women’s active role in governance and decision-making. Together, these elements outline practical entry points for enabling vulnerable women to participate more effectively and equitably in seed systems. The term “vulnerable women farmers” refers in this study to women with limited financial resources and reduced capacity to independently access agricultural inputs. In the Malian context, programs often identify such women through their participation in community-based savings and loan groups (Tèkèrèninw), which are self-organized by women who are financially vulnerable. As our intervention also engaged women from these groups, we used the term “vulnerable women farmers” to describe the target population.

To illustrate how farmers access seeds through different channels, Box 1 outlines the formal, informal, and integrated/community-based seed system pathways, highlighting entry points for inclusivity.

BOX 1 **Inclusive seed system pathways.**

**Seed Pathways (3 routes):**

- **Formal:** Breeding → Seed Companies → Agro-dealers → Farmers
- **Informal:** Farmer-saved seed → Exchanges → Local markets → Farmers
- **Community-based:** Research/Extension → Seed banks/cooperatives → Farmer groups → Farmers

**Inclusivity Components (applied across all pathways):**

- **Access:** Targeted training for women, recognition of traditional seed keepers
- **Affordability:** Subsidies, micro-credit, community savings groups
- **Physical Accessibility:** Local distribution, reduced travel burden, women-led seed outlets
- **Participation:** Women in governance of seed banks, cooperatives, and decision-making bodies

**Source:** Authors own elaboration.

## Insights from inclusive seed systems campaigns

Women in seed production face multiple challenges that limit their participation in formal seed systems. To address this, the ICRISAT Gender Program, in collaboration with the IER/ECOFIL Program, launched a targeted seed distribution campaign in Bla and Koutiala districts during 2020 farming season. The initiative provided vulnerable women farmers with early and medium-maturity millet and sorghum varieties, improving their access to climate-resilient seeds and strengthening their role in seed-to-nutrition pathways. The “seed-to-nutrition pathway” denotes an integrated framework that connects the development, dissemination, and adoption of improved, nutrient-dense crop varieties with downstream processes such as cultivation, post-harvest handling, value addition, and consumption, aimed at enhancing dietary quality and nutritional outcomes.

Despite these efforts, women’s participation in formal seed production remains low, with only 7.47% cultivating improved sorghum and 7.28% growing improved millet. Predominant reliance on informal seed sources highlights the need for further interventions (8–10). Table 1 presents the sorghum and millet varieties cultivated by women farmers in the study areas. For sorghum, both hybrids (Pablo, Sewa, and Fadda) and open-pollinated varieties (Soumba, Tiebile, Bobodje, Djiguifa) are used alongside several local landraces (Seguetana, Niobleni, Bandoga). In contrast, millet cultivation relies more on local varieties (Souna, Sanioba) with limited adoption of improved open-pollinated varieties such as Guéfoué 16, Sanioteli, and Toroniou, the latter being the only improved variety reported. This pattern reflects women’s reliance on a mix of hybrids, OPVs, and local seeds in sorghum, while millet continues to be dominated by traditional landraces with minimal penetration of improved materials. The intervention, implemented across nine villages, targeted women farmers who met clearly defined selection criteria. A total of 57 women were selected based on their ownership of individual plots and their voluntary commitment to cultivate certified crops. In addition to land ownership and willingness to participate, other considerations included prior farming experience, interest in growing sorghum and millets, and the capacity to manage crop cultivation independently. These criteria were crucial for ensuring active participation and responsible use of resources. As part of the initiative, 126 kg of certified seeds—107 kg of sorghum and 19.6 kg of millet—were distributed to the selected farmers. Seed allocations ranged from 200 grams to 500 grams per women farmer for millet and 400 grams to 5,000 grams for sorghum. Women received three sorghum varieties: *Seguifa* (100-day maturity), *Soubatimi* (110-day maturity, dual-purpose), and *DT-15*, and five millet varieties: *Synthétique 00–02*, *Synthétique 03–03* (78-day maturity), *Mil aristé* (72-day maturity), *Synthétique 00–06* (80-day maturity), and *Maiwa* (82-day maturity).

**Table 1:** Crop varieties cultivated in the selected locations.

Crop	Varieties
Sorghum	Pablo (Hybrid), Sewa (Hybrid), Fadda (Hybrid), Soumba (Open pollinated variety-OPV), Tiebile (Open pollinated variety-OPV), Bobodje (Open pollinated variety-OPV), Seguetana (local), Niobleni (local), Bandoga (local)
Millet	Guéfoué 16 (Open pollinated variety-OPV), Sanioteli (Open pollinated variety-OPV), Souna (local), Toroniou* (Open pollinated variety-OPV), Sanioba (local), Djiguifa (Open pollinated variety-OPV)

*Toroniou is the only improved millet variety cultivated among those listed.

The selection of early- and medium-maturing varieties was a strategic response to increasing climate variability, particularly the shortening of growing seasons and delayed rainfall onset in southern Mali (11, 12). Early maturing varieties help mitigate the risk of crop failure due to late rains or early cessation of the rainy season, while medium-maturity varieties balance yield potential with adaptability (13). Prioritizing early and medium-maturing varieties helped mitigate climate risks and strengthened women’s participation in seed systems. While research has provided insights into variety adoption, significant knowledge gaps remain regarding women’s roles in seed markets, particularly in the production and distribution, due to limited technical knowledge and domestic constraints (14, 15). These findings highlight the importance of complementing seed distribution efforts with capacity building, gender-responsive extension, and institutional support to enable women’s sustained engagement in seed systems.

While improved sorghum varieties are more commonly cultivated than improved millet varieties, women face significant challenges in accessing high-quality seeds. Limited financial resources at the time of sowing often prevent them from purchasing improved seeds, despite their availability in the market. Moreover, the physical distance of seed sale points from production areas further restricts women’s access to improved varieties. These barriers highlight the need for targeted interventions that improve women’s access to quality seeds through financial support mechanisms, localized seed distribution networks (16), and policy measures that facilitate timely access to inputs.

Several initiatives are addressing this gap. For example, the World Food Programs resilience program provides cash transfers to vulnerable communities, particularly women in conflict-prone regions, enabling them to invest in livelihood-improving inputs such as seeds and tools. Other programs focus on building financial self-reliance by training and supporting women to create and manage community-based savings and loan groups known as *Tèkèrèninw*. These informal microfinance systems offer a practical and culturally appropriate way for women to access funds during the planting season, thereby facilitating timely access to inputs and improving their participation in seed systems. Expanding such models—complemented by policy support and market linkages—can significantly strengthen women’s role in resilient seed systems and food production. Further, streamlining processes is essential to ensure funds are released on time, especially before the planting season. Timely support enables better access to inputs and improves participation in seed systems.

Women primarily access improved sorghum and millet seeds through informal networks, with relatives serving as the dominant source for 41.17% of farmers. This reliance is more pronounced for millet (52.65%) than sorghum (29.70%), indicating that traditional family-based seed-sharing systems play a crucial role in sustaining millet cultivation. Exchange and barter systems contribute to 20.00% of seed sources, with a slightly higher prevalence for millet (21.35%) than sorghum (18.75%), reflecting the continued importance of reciprocal farming practices in rural communities. Farmer cooperatives provide an alternative source of improved seeds for 8.35% of women, with greater uptake for millet (12.00%) than sorghum (4.70%), suggesting that cooperatives could be leveraged more effectively to integrate women into formal seed systems.

While these findings demonstrate the resilience and social embeddedness of informal seed networks, they also raise important concerns regarding seed quality, traceability, and access to a broader range of improved varieties. The continued reliance on non-commercial channels may limit the diffusion of high-performing, climate-resilient seeds and pose challenges for maintaining genetic purity and varietal turnover (17). Strengthening the interface between informal and formal systems—particularly through gender-responsive cooperatives and decentralized seed enterprises—could help address these limitations while respecting local practices and knowledge systems.

Despite these informal networks, formal seed channels remain underutilized. Only 2.77% of women purchase seeds from agro-dealers or seed cooperatives and companies, and just 1.77% receive seeds through extension services, with slightly higher access for millet (2.00%) than sorghum (1.55%). The low adoption of formal seed markets reflects economic constraints, limited awareness, and a lack of gender-responsive extension services. Community leaders play a minor role in seed distribution, particularly for sorghum (3.10%), with no reported influence on millet seed access, highlighting gaps in localized leadership engagement in formal seed dissemination.

Seed recycling is widespread, with 66% of women reusing sorghum seeds before replacement. The average replacement interval for improved sorghum seeds is approximately 7 years—significantly exceeding the recommended period of 2 to 3 years (18). In some cases, farmers continue using the same seed stock for up to 8 years. This extended recycling period is largely driven by limited access to formal seed markets, financial constraints, and inadequate awareness of the agronomic benefits of timely seed renewal. However, prolonged use of the same seed stock raises critical concerns regarding declining seed vigor, yield potential, and increased risk of genetic erosion (2, 19). Over time, recycled seeds may lose their adaptive traits, accumulate pathogens, and exhibit greater variability in plant performance, ultimately undermining productivity and resilience.

For millet, the average seed replacement cycle is slightly shorter at 5.5 years, compared to the national average of 5 years. This may be attributed to the relatively recent introduction of improved millet varieties in Mali—most of which have been developed over the past 25–30 years, in contrast to the longer breeding history of sorghum. Additionally, in regions like Koulikoro, Dioila, and Koutiala, the vulnerability of traditional millet varieties to erratic rainfall and bird predation has prompted a gradual shift among farmers over the last decade toward improved short- and medium-duration varieties better suited to changing climatic conditions (12, 20). The extended use of recycled seed underscores the urgent need for awareness campaigns and gender-responsive seed system interventions that promote timely seed replacement while preserving access to genetically diverse and high-quality materials. These findings highlight the need for gender-inclusive interventions in seed systems. Improving women’s access to high-quality seeds, increasing awareness about the importance of timely seed replacement, and strengthening formal seed distribution channels are key priorities. Addressing these gaps is essential to boost crop productivity, maintain seed quality, and support the long-term sustainability of millet and sorghum agri-food systems in Mali.

## Linking seed systems to nutrition pathway

Under the World Food Program (WFP)-funded project, ICRISAT developed a crop variety catalogue as part of the Integrated Resilience Package and its nutrition component. This catalogue serves as a key resource for farmer organizations, development agents, and institutions, guiding the selection of climate-resilient and nutrient-dense crop varieties suited to the project implementation zones agroecological conditions.

The catalogue features 36 improved varieties of millet, sorghum, groundnut and cowpea developed through crossbreeding and multi-year evaluations. The varietal selection process was based on multiple scientific and context-specific criteria to ensure relevance and impact. These included agroecological adaptability—particularly annual rainfall patterns—drought tolerance, and growth duration (short to medium cycle), which are critical for performance in rainfed farming systems. In addition, varieties were prioritized for their biofortification with essential micronutrients (such as iron and zinc), dual-purpose use (grain and fodder), and post-harvest processing suitability to enable transformation into marketable food products. This multi-criteria approach ensured the inclusion of varieties that are not only agronomically robust but also nutritionally beneficial and economically viable, supporting the development of resilient and nutrition-sensitive food systems in Mali. By integrating seed-to-nutrition pathways into inclusive food systems, this initiative enhances agricultural productivity and addresses malnutrition. The catalogue provides essential agronomic and nutritional details, including genetic type, growth cycle, yield potential, resistance to pests and diseases, and fortification type, supporting the adoption of improved varieties for resilient and nutrition-sensitive food systems in Mali. A total of 5 millet varieties, 10 sorghum varieties, 11 groundnut varieties and 8 cowpea varieties have been identified for introduction in the project communities. Their characteristics are presented in Table 2, providing a foundational resource for scaling resilient and nutrient-dense crop production in Mali.

**Table 2:** Catalogue of fortified and climate-resilient crop varieties.

Variety name	Crop/genetic type	Plant height (m)	Sowing to 50% maturity (days)	Grain yield potential (t/ha)	Resistance to diseases, pests, and weeds	Isohyets (mm)	Fortification (type)
Chakti	Pearl Millet/OPV	1.60–1.80	47–50	1.30–1.50	Resistant to downy mildew, drought-tolerant, Striga-tolerant	300–550	Fe (60 ppm) and Zn (42 ppm)
ICMV 167006 (ICRI-Tabi)	Pearl Millet/OPV	1.8–2.2	50–55	1.5–1.7	Resistant to downy mildew, drought-tolerant, Striga-tolerant	300–600	Fe (47 ppm) and Zn (35 ppm)
ICMP 177002*	Pearl Millet/OPV	1.70–1.85	45–50	1.4–1.7	Resistant to downy mildew, drought-tolerant, Striga-tolerant (tested in farmer fields)	300–600	Fe (68 ppm) and Zn (46 ppm)
ICMP 207555*	Pearl Millet/OPV	1.70–1.85	45–50	1.4–1.7	Resistant to downy mildew, drought-tolerant, Striga-tolerant (advanced trials)	300–600	Fe (85 ppm) and Zn (55 ppm)
ICMP 187038*	Pearl Millet/OPV	1.8–2.2	50–53	1.6–1.8	Resistant to downy mildew, long panicles, drought-tolerant, Striga-tolerant (tested in farmer fields)	300–600	Fe (51 ppm) and Zn (34 ppm)
ICGV 93437	Groundnut	NA	85–90	1.5–2.5	Drought-tolerant, early-maturing	300–600	>50% oil, up to 28% protein
ICGV-IS 131054	Groundnut	NA	85–90	1.5–2.5	Early-maturing, drought-tolerant	300–600	>50% oil, up to 28% protein
ICGV-IS 131079	Groundnut	NA	85–90	1.5–2.5	High-yielding, foliar disease-tolerant	450–1200	>50% oil, up to 28% protein
ICGV-IS 131085	Groundnut	NA	90–95	1.5–2.5	High-yielding, foliar disease-tolerant	450–1200	>50% oil, up to 28% protein
Jakunbe	Sorghum/OPV	3.5	60	2	Drought-tolerant	400–800	High protein (>14%), Fe (>60 ppm), Zn (>30 ppm)
Seguifa	Sorghum/OPV	2	60	3	Striga-tolerant	400–800	Dual-purpose, high in carbohydrates/energy (>85%)
Fadda	Sorghum/Hybrid	3	85	4.5	Mold-tolerant	800–1200	Hybrid, dual-purpose, high in Fe (>60 ppm), high energy (>85%)
Acar 1	Cowpea/OPV	Semi-erect	65–70	1.5–2	Drought- and Striga-resistant	400–800	High in protein (27.81%)
Simbo	Cowpea/OPV	Semi-erect	65–70	1.5–2	Drought- and Striga-resistant	400–800	High in protein (28%)

Source: The crops listed in the Catalogue under the UN WFP project, 2021. *Varieties are at different stages of testing and not yet released for commercial cultivation. OPV, Open-pollinated variety.

**Figure 1:**
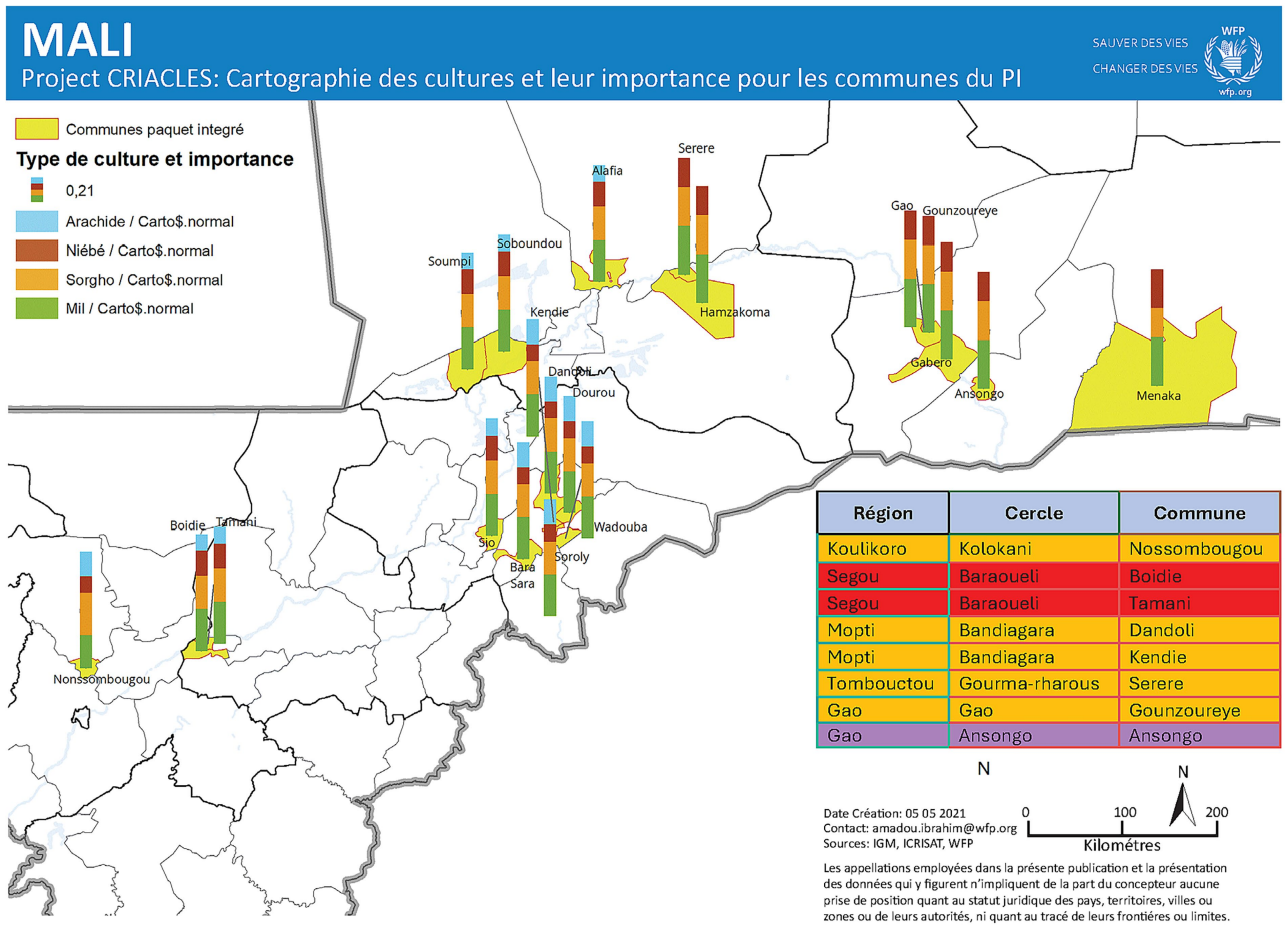
Mapping of crops and their importance for the selected communes in Mali. Arachide = Groundnut; Niébé = Cowpea; Sorgho = Sorghum; Mil = Pearl millet. Reproducted from ICRISAT & WFP (21) with permission.

A comprehensive crop mapping exercise was carried out across 20 municipalities to assess the relevance and distribution of four key crops: millet, sorghum, groundnut, and cowpea (Figure 1). The analysis aimed to identify varieties best suited to each agroecological zone, taking into account the growth cycles of improved varieties developed through research and the region’s prevailing rainfall patterns. The mapping combined the use of GIS tools with rural participatory appraisals and focus group discussions with men and women farmers in the selected regions, ensuring both spatial accuracy and a nuanced understanding of local cropping patterns, preferences, and constraints. A multi-faceted approach was implemented to raise awareness and encourage the adoption of improved crop varieties, combining traditional outreach with modern communication strategies. Large-scale field demonstrations showcased high-yielding, climate-resilient varieties alongside improved agronomic practices such as seed treatment with Apron-Star, optimal planting densities, and targeted fertilizer use. A participatory protocol, developed with field partners, guided the systematic monitoring of these demonstrations. Complementary training sessions equipped farmers with skills in crop production and post-harvest management to support sustained adoption.

Preliminary feedback from participating communities suggests a positive response. Farmers reported improved germination rates, higher yields, and better understanding of fertilizer and spacing techniques. In some sites, farmers expressed interest in procuring seed for the following season, indicating early signs of uptake. While comprehensive impact evaluation is ongoing, these initial observations underscore the potential of integrated demonstration and training efforts to influence adoption behavior.

Women’s access to quality seeds is shaped by a combination of traditional practices and new interventions. Many women continue to source seeds from previous harvests, as this practice is deeply rooted in local farming systems and ensures resilience, but such reliance often restricts exposure to improved varieties. Where local stocks are insufficient, women may need to purchase seeds; however, affordability poses a challenge given their limited financial resources. Interventions therefore work to reduce cost barriers through subsidies, group purchases, cooperatives, and community-based savings and loan groups (Tèkèrèninw). Physical accessibility also remains a constraint, with formal seed outlets often located far from rural communities. To address this, strategies such as village-level seed shops, community seed banks, mobile distribution, and farmer cooperatives are promoted to bring seeds closer to women farmers. Together, these pathways highlight that inclusive seed systems require not only the introduction of improved varieties but also deliberate efforts to ensure that women can access them in affordable and physically feasible ways, while still retaining valued local practices.

## The role of biofortified crops in improving nutrition

The transformation of agricultural outputs into consumer-ready products depends on resilient and inclusive seed systems that underpin productivity, food processing, and nutrition security. For biofortified crops, value addition is a crucial step in enhancing their adoption, improving nutritional outcomes. Processing nutrient-rich crops like high-iron millet into flours, snacks, and fortified foods tailored to regional diets helps translate agronomic gains into tangible health benefits. Product development further strengthens this process by creating culturally accepted items such as porridge, biscuits, semolina mixes, and weaning foods—enhancing consumer acceptance and market demand. Strengthening supply chains through direct linkages among farmers, processors, retailers, and institutional buyers facilitates the scale-up and market integration of biofortified products. At the same time, consumer awareness and demand creation—through targeted behavior change communication—play a critical role in promoting the health benefits of these foods and encouraging widespread adoption.

Finally, policy and institutional support are essential for sustaining impact. The integration of biofortified foods into school feeding programs, public health initiatives, and national nutrition policies helps embed these innovations within broader food systems. Streamlining and reinforcing each stage of the value addition process is key to improving access to nutritious foods, advancing dietary outcomes, and building more resilient, inclusive food systems.

## Case study: women’s entrepreneurship in conflict zones

Enhancing women’s participation in the seed-to-nutrition pathway requires an integrated approach that builds capacity, improves financial access, fosters market integration, and supports the adoption of appropriate technologies. A compelling example of this can be seen in Central Mali, where Fatima (name changed for confidentiality) and her women’s group are translating improved seed access into tangible nutrition and livelihood outcomes. Their work illustrates how community-led initiatives can leverage biofortified crops to drive inclusive and sustainable development.

Using Chakti—a high-iron and zinc-rich millet variety developed by ICRISAT in partnership with HarvestPlus and released in Niger in 2018—the group produces ready-to-cook and ready-to-eat products tailored to local dietary preferences. Chakti has since been introduced across several West African countries, including Mali, Burkina Faso, Ghana, and Senegal, due to its resilience in semi-arid regions and its potential to combat micronutrient deficiencies such as anemia. This initiative exemplifies the downstream impact of inclusive seed systems when paired with effective processing, marketing, and nutrition education. In more fragile regions like Gao, Tombouctou, Segou, and Bandiagara, similar women-led enterprises have emerged as models of resilience and innovation. Despite challenges posed by insecurity and political instability, these women have built thriving businesses that improve local nutrition and contribute to household and community resilience. They source biofortified grains and combine them with locally available staples such as rice, groundnut, millet, and cowpea to develop nutrient-rich, culturally accepted products—including millet flour, semolina, doughnuts, and peanut butter. These products are distributed to school canteens and health centers, directly supporting child nutrition and dietary diversity.

Beyond food production, these groups also foster financial self-reliance through thrift groups that save at least XOF100,000 (approximately US$162) monthly from profits, reinforcing both economic empowerment and social cohesion. Saving XOF 100,000 per month per thrift group is significant in the context of rural Mali, where many households live on less than US$2 per day. For comparison, this monthly saving is equivalent to more than 2 months’ income for a typical rural household. It can cover essential expenses such as school fees, healthcare, and food for an entire family, highlighting the substantial economic impact of these women-led initiatives on household financial stability and resilience.

This holistic model—rooted in improved seed access, local food processing, and community-based financial systems—demonstrates how women’s leadership can transform seed-to-nutrition pathways into engines of sustainable development, particularly in fragile and conflict-affected settings.

## Conclusion

Empowering women in the seed-to-nutrition pathway is critical to building inclusive seed systems and resilient food systems in Mali. Providing women with technical training in seed selection, multiplication, and storage—alongside access to financial services and strengthened market linkages—can help them move from passive recipients to active drivers of seed production and value addition. This not only enhances the sustainability and competitiveness of millet and sorghum value chains but also contributes to improved household nutrition and livelihoods.

To scale and replicate these efforts, several practical steps should be prioritized by governments, NGOs, and development partners:

- Invest in gender-responsive extension services that provide tailored training on improved seed production, agronomic practices, and food processing technologies.
- Expand access to inclusive financial services, such as group savings schemes, microcredit, and seed vouchers, to support women’s investment in inputs and small-scale enterprises.
- Strengthen women-led cooperatives and enterprises by supporting their formal registration, capacity development, and direct linkages with institutional buyers and markets.
- Leverage digital tools—including mobile-based platforms for weather forecasting, market information, and digital payments—to enhance women’s decision-making and market access.
- Integrate biofortified crops into public procurement programs, such as school feeding and health initiatives, to create stable demand and reinforce nutrition-sensitive food systems.
- Support policy frameworks that recognize and promote women’s roles in seed systems and agri-food value chains, including land tenure security and representation in decision-making bodies.

A coordinated, multi-sectoral approach that combines technical support, financial inclusion, market development, and policy advocacy is essential to unlock women’s full potential as agents of agricultural transformation, food security, and climate resilience.

## Recommendations

To build inclusive food systems, it is essential to strengthen the seed-to-nutrition pathway by ensuring equitable access to quality seeds, enhancing women’s participation in seed systems, and integrating nutrition-sensitive agricultural practices. Expanding women’s role in seed production can create a more resilient and diversified food supply while addressing malnutrition. Efforts should focus on improving access to biofortified and climate-resilient seeds through targeted distribution programs, community seed banks, and cooperative seed enterprises led by women. Additionally, inclusive seed systems must be linked to sustainable food processing and value addition, enabling women to participate in the full spectrum of the seed-to-nutrition pathway, from production to market-ready nutritious products.

Capacity-building programs should be expanded to equip women farmers and processors with technical expertise in seed selection, multiplication, storage, and processing techniques. Access to finance remains a significant barrier; therefore, facilitating microloans, cooperative funding mechanisms, and public-private partnerships can enhance women’s investment in both seed systems and value-added food enterprises. Furthermore, strengthening seed-to-market linkages through digital tools, e-commerce platforms, and direct procurement models can increase the affordability, availability, and desirability of biofortified food products in inclusive food systems.

To enhance the seed-to-nutrition pathway, policies must promote the integration of biofortified crops into public food and nutrition programs, such as school feeding initiatives and maternal health interventions. This requires multi-stakeholder collaboration, including government agencies, research institutions, and development organizations, to create an enabling environment for women-led agribusinesses. Additionally, community-based nutrition education should be embedded within seed distribution initiatives to increase awareness of the benefits of biofortified foods and drive consumer demand.

By adopting a seed-to-nutrition pathway approach, Mali can advance gender-responsive, climate-smart agriculture that ensures smallholder farmers, particularly women, are not only seed recipients but active participants in shaping inclusive food systems. This holistic strategy will contribute to improved dietary diversity, economic empowerment, and sustainable food systems.

## Data Availability

The raw data supporting the conclusions of this article will be made available by the authors, without undue reservation.
